# Corrigendum to “Ethical language and decision-making for prenatally diagnosed lethal malformations” [Semin Fetal Neonatal Med 19 (5) (2014) 306–311]

**DOI:** 10.1016/j.siny.2014.10.007

**Published:** 2015-02

**Authors:** Dominic Wilkinson, Lachlan de Crespigny, Vicki Xafis

**Affiliations:** a Oxford Uehiro Centre for Practical Ethics, Faculty of Philosophy, University of Oxford, Oxford, UK; b Department of Obstetrics and Gynaecology, University of Melbourne, Blairgowrie, Victoria, Australia; c Robinson Institute, Discipline of Obstetrics and Gynaecology, University of Adelaide, Adelaide, South Australia, Australia

The authors wish to draw readers' attention to an incorrect figure in Table 1 of the above review.

The authors would like to apologise for any inconvenience caused.

Under the column headed '**Probability of live birth (in absence of termination)**', the fetal survival rate for Trisomy 13 should read **46-51%** and not 28-46%, as originally printed.

**Table 1:** Published outcome for severe congenital anomalies frequently described as lethal.^a^

Severe congenital anomalies	Prevalence	Probability of live birth (in absence of termination)	Median postnatal survival	Proportion surviving >1 week/>1 year	Longest reported survivals
Renal agenesis	1.7/10,000 [23]	Not reported	<24 h [23]	<5%	13 months [97,98]
Anencephaly	10/10,000 pregnancies	62–72% [25,26]	<24 h [26,27]	0-14%>1 week/7% >1 year [18,29]	10 months [30]
	2.6/10,000 births [24]		55 min [28]		2.5 years [31]
Thanatophoric dysplasia	0.4/10,000 [32]	Not reported	Not reported	Not reported	5 years [33]
					9 years [34]
Trisomy 18	2.6/10,000 [24]	48–51% [35,36]	14 days [37]	35–65%>1 week/14–19% >1 year [18,29]	27 years [38]
					30 years [39]
					50 years [40]
Trisomy 13	1.2/10,000 [24]	46–51%[35,36]	10 days [37]	45-57%>1 week/14–21% >1 year [18,29]	19 years [41]
					27 years [42]
Holoprosencephaly	0.5/10,000 [43]	Not reported	4–5 months [44]	71%>1 week/ 47% >1 year [29]	6 years [45]
					11 years [44]
					13 years [43]
					19 years [46]

a Using recent population cohort studies where available.

